# “Hi Mr belly” - A personal perspective on the obesity crisis and how to tackle it

**DOI:** 10.1016/j.amsu.2019.07.009

**Published:** 2019-07-09

**Authors:** Emre Pakdemirli

**Affiliations:** West Hertfordshire Hospitals NHS Trust, Waverley Rd. St. Albans, AL3 5PN, UK

**Keywords:** Obesity, Exercise, Body mass index

Once, whilst I was taking a leisurely stroll up the slope a short distance from my neighbourhood, I heard a child––one of a group of seven or eight kids aged perhaps 10 or 11 years––chanting at me from the other side of the street: ‘Hi Mr. Belly! Hi Mr. Belly!’ I turned my head toward him the second time that he called, but I did not respond to him, as I felt a mixture of feelings ranging from offended to slightly bemused. However, when I replayed the whole incident in my mind, I realised that the boy only meant a bit of mischief rather than a verbal assault. I turned away with a concealed smile and told myself, ‘Yes, he is right! He is absolutely right’. Since I am a physician (a radiologist), I should not have the body shape that I have. Yet, the children did not know I am a doctor. Had they known, perhaps they would have teased me even more. I walked down the slope and headed towards my home with a big smile on my face. I then shared my experience with my family WhatsApp group. If this manuscript is published, I will definitely share that experience with my colleagues via our WhatsApp group too.

I am 53 and overweight. Actually, I am on the verge of morbidly obese, with a body mass index (BMI) of near 40. I have never smoked or consumed alcohol in my entire life. Obese, yes, but otherwise healthy and not taking any medication. My cholesterol, LDL, HDL and blood glucose levels are within normal ranges. My LFTs nearly doubled as I have known fatty liver, but LFTs have been stable for well over 30 years.

However, I know belly fat is a killer, and I should do something about it. That's why I have recently been trying to increase my steps. I have a smartphone app that counts steps. I initially set up my daily step goal as 7500 steps, which is less than the recommended 10,000 per day. With an exception of well over 10,000 steps some days of the week, overall, I have trouble achieving 7500 steps in a week.

Is there a science behind 10,000 steps per day? Perhaps brisk walking at a faster pace with fewer steps would be more beneficial than simply pratting about. Yet, step-counting has been widely accepted in physical activity interventions. A cadence of more than 100 steps per minute is a heuristic threshold value indicative of absolutely defined moderate-intensity ambulatory activity in healthy adults [1]. At the same time, obesity is a well-known chronic condition affecting 27% of the UK adult population. Ischemic heart disease, type 2 diabetes and certain cancers are attributable to excessively high BMI [2].

I remember asking for health advice about losing weight from my GP. Apart from receiving some well-known cliché advice, I was never put on a monitoring programme, and I remained off from the radar, as only 42% of obese adults who receive proper weight management advice in the UK [3].

On the other hand, if I carried on a slightly accelerated walking pace, I would eventually achieve ship-shape condition, lose some weight, and decrease my BMI in a couple of years. However, I would have to lose my nickname of ‘Mr. Belly’.

## 1 Provenance and peer review

Not commissioned, internally reviewed.

## Ethical approval

No Ethical Approval needed.

## Sources of funding for your research

No funding.

## Author contribution

Dr.Emre Pakdemirli is the only author in this piece.

## Consent

Not needed.

## Conflicts of interest

No conflict of interest.

## Registration of research studies

1. Name of the registry: Not applicable.

2. Unique Identifying number or registration ID:

3. Hyperlink to the registration (must be publicly accessible):

## Guarantor

Dr.Emre Pakdemirli.
